# Mentalization-based approach for schizophrenia spectrum disorders: a psychotherapeutic proposal for evolved schizophrenic trajectories and serious mental disorders

**DOI:** 10.3389/fpsyt.2024.1240393

**Published:** 2024-05-08

**Authors:** Pedro Sanz, Nuria Tur, Fernando Lana

**Affiliations:** ^1^ Programa de Trastorno Mental Grave, Area de Gestión Clínica de Psiquiatria y Salud Mental (AGCPSM), Hospital 12 de Octubre, Madrid, Spain; ^2^ Servicio de Psiquiatria, Unidad del Niño y Adolescente, Hospital Clínico San Carlos, Madrid, Spain; ^3^ Instituto de Neuropsiquiatría y Adiciones (INAD), Centro Emili Mira y Hospital del Mar, Parc de Salut Mar, Barcelona, Centro de Investigación en Red de Salud Mental (CIBERSAM), Departamento de Psiquiatría, Universidad Autónoma de Barcelona, EspañaIMIM (Hospital del Mar Medical Research Institute), Barcelona, Spain

**Keywords:** mentalization-based treatment, schizophrenia, schizophrenia spectrum disorder, serious mental disorder, psychotherapy process, self-agency, dyadic

## Abstract

There is a growing interest in psychotherapeutic approaches to pre-psychotic high-risk states or first-episode psychosis, where mentalization-based treatment has shown its utility. This article presents a mentalization-based approach for the treatment of those individuals diagnosed with an evolved schizophrenia spectrum disorder, whose characteristics make them especially inaccessible to reflective psychotherapeutic treatment. A synthesis of the conceptual frameworks that justify the needs for technical modification of the mentalization-based treatment foundational techniques is carried out, followed by the proposal of adaptations, with a focus in self-agency and patient-therapist dyad. Therapeutic interventions are outlined, including illustrative examples. The mentalizing approach presented here holds promise for future research and treatment opportunities for patients with evolved schizophrenia and other serious mental disorders.

## 1 Introduction

Schizophrenia, one of the world’s top 15 leading causes of disability (1), is a complex disorder with multifactorial pathology. It is associated with reduced social connections, lower employment rates, and impaired ability to live independently (2).

Schizophrenia has a dual etiopathogenesis, combining both neurodevelopmental and acquired factors (3, 4), where each individual’s presentation of the disorder is influenced by genetic predisposition and specific biographical or environmental factors. These alterations manifest at various levels, ranging from neurobiological to sociocultural aspects (5). Schizophrenia and Schizophrenia spectrum disorders (SSM) (6), can affect several neuropsychological functions, including volition, cognition, affect, and psychomotor abilities (7). It also impairs metacognitive and social cognitive functions (8) and, specifically, mentalizing functions (9). These impairments prevent patients from adapting to their environment, causing significant distress and lower functional performance than expected socially and culturally (10).

Antipsychotic drugs have proven to be the treatment with the best evidence level (11), but despite its availability, research indicates that disability among patients with evolved schizophrenia has shown little improvement over the past century (12) In recent years, the focus has been shifting to the prevention, detection and diagnosis of prodromal forms or the initial phases of the disease (first psychotic episodes) (13, 14), while early psychosocial treatments, psychotherapies and trainings have also been developed (15–17) improving evolution and outcome (16).

Mentalization-based treatment (MBT) is an empirically validated psychotherapeutic approach for serious mental disorders, such as personality disorders, which reduces symptoms and improves social functioning (18, 19). Research projects are currently underway for MBT in psychosis (20–25), with preliminary reports suggesting its efficacy in first-episode psychosis and SSD (26–29). Promising research is also ongoing regarding clinical high-risk states for psychosis (30) and emerging psychosis (31, 32).

The approach presented here aims to adapt MBT foundational technique to individuals with evolved schizophrenia spectrum disorders with multiple episodes, persistent severe psychotic symptoms or neurocognitive dysfunctions. The interventions are tailored according to the patient’s mentalization and agency capacities observed in the therapist-patient dyad.

We will first describe the conceptual framework that will guide our proposal for the adaptation of MBT to individuals with evolved SSDs. To understand the special characteristics of the psychotherapeutic relationship with patients with severe mentalization problems, we will turn to the postulates of Friston’s free energy minimization and predictive coding model and the Second-person neuroscience paradigm, which provide us with neuroscientific foundations to understand the creation of self-boundaries and the configuration of dyadic relationships. From that understanding, we will then rely on Gergely’s theory of self-agency development, which will serve as a guide to tailor our psychotherapeutic interventions.

## 2 Neuroscientific foundations

Friston’s neuroscientific model integrates Feynman’s principles of free energy and Bayesian probabilistic inference (33, 34). Free energy principles posit that a feature of biological systems is to maintain stability and form when faced with continually changing environment. This preservation of order is referred to as homeostasis. In self-organizing systems, homeostasis is governed by the organism’s phenotype. The model suggests that organisms can tolerate limited levels of internal “disorder” to sustain low free-energy levels, as higher levels would lead to instability and risk. According to this perspective, biological success lies in minimizing the free energy, regulating oneself against the environment and influencing the environment to align with one’s interests.

To achieve this, biological agents must anticipate and cover against a range of environmental changes in order to maintain stability in the face of a variety of conditions. These are known as “predictions” in Bayesian probabilistic terms (35), and any deviation from the expected predictions caused by the environment is referred to as “predictive error” or “surprise”. Thus, to maintain internal equilibrium, predictive errors should be minimal and predictions should be accurate, being interpreted as certainties or confirmations, and allowing us to efficiently manage the environment (35).

In the case of the central nervous system and its connections as a self-organizing system, it must adhere to these principles to maintain functional viability (33). Afferent and efferent states within the central nervous system should remain within physiological limits. Thus, in accordance with this model, it is suggested (33) that the cerebral cortex does not generate “orders” as traditionally believed but generates “predictions.” And sensory receptors, in turn, transmit “prediction errors” (34, 36).

It is hypothesized that the central nervous system contains representations of itself in relation to the environment, which is generated and then expanded during its development (37). At this level, these representations exhibit a hierarchical structure, increasing in associative capacity and complexity as we move from basic to more developed structures and from a lower to a higher layer of the cerebral cortex (38–40).

According to this theoretical model (33, 35, 41) the central nervous system can minimize its prediction error in two ways: The first is by changing or expanding its predictions to align more closely with the sensory input, that is, giving value to the predictive error and modifying the prediction, thereby generating more complex mental representations (“learning”). This would occur through a “bottom-up” regulation. Once a better hypothesis has been established to explain the cause of sensory stimulation, instability is attenuated and the rest of the hypotheses are excluded. The second way is by attenuating or disregarding predictive errors, giving greater weight to the predictions made by the cerebral cortex over the information received from the sensory receptors. This is a “top-down” regulation and involves changing the sampling of the environment to confirm the predictions, which may imply an outward-facing motor action. In predictive terms, we can hypothesize that a tolerable level of predictive error triggers contemplative (bottom-up) or exploratory (top-down) curiosity. However, excessive predictive error will lead to saturation, failed responses to the environment, and a collapse in the predictive system.

The Bayesian probabilistic model implies a bidirectional relationship. The probabilistic link between prediction and predictive error suggests that both factors influence and condition each other. This provides adaptive advantages in relation to the environment, helping the organism to determine its “limits” and where its “epithelium” or friction zone is located (35, 41). From this understanding, we can hypothesize that the only possibility an agent has to “know” its state at any given moment is through the inseparable connection between itself and the world that it is capable of representing to itself through its sensory observations, self-generating a model that continually tests, remakes, and expands itself, thereby remaking and expanding its self-image in relation to the environment. The “Markov blanket” (41, 42) concept represents a probabilistic model to represent this, defining a variable based on the set of variables with which it is exclusively related, while disregarding unrelated ones. In our hypothesis, we adopted this model to define the self-boundaries. Depending on the representations that “descend” and the sensory impressions that “rise,” analogous to a radar pulse bouncing off an object to determine its position, individuals can generate a self-image and respond to the environment (41).

### 2.1 Dyadic systems and second-person neuroscience

In the context of the agent-environment relationship, we can also consider a unique scenario when the “environment” is another agent like ourself, that is, when a being enters into a relationship with another being, thereby both their inferential systems come into play (43). This gives rise to a dyadic inference system, where each member of the dyad acts as both the emitter and receiver of predictions and predictive errors (35, 44). This dyadic system, with its distinct characteristics, can be seen as a foundational element in all psychotherapies.

EXAMPLE

*When a patient receives a predictive error in the form of an ambiguous or unexpected response from the therapist, such as a comment or facial expression that this particular patient does not understand, or interprets as hostile, it will generate discomfort in his predictive system, and he will have to manage it.*

*In such cases, the patient can escalate the situation to a higher level of representational complexity, for example, by not remaining fixated on the concrete and instead adding more representations, such as recognizing the stable, constant, and reliable relationship that he have with his therapist, and make another prediction like, “Ah, it’s irony. He’s using humor.” This new prediction dissolves the discomfort caused by the predictive surprise, exemplifying a bottom-up regulation. But let us imagine that the patient’s arsenal of predictions is insufficient to deactivate this unexpected response, since the last prediction he makes, such as “He’s tired of me. He thinks I’m stupid,” does not attenuate that “free energy,” and the individual finds himself unstable in the face of the environment, uncomfortable in the dyad with his therapist. In such cases, the patient can expand their perceptual sampling to try to “adjust” it to his expectations (“Is it true or not true that my therapist thinks I am stupid?”). For example, they might observe the therapist’s expression in greater detail to look for indications that could resolve his doubt, or he could emit a stimulus to the dyad, such as asking a question to the therapist like, “I did not understand the last thing you said. What did you mean?” These actions aim to decrease the predictive error at its source and alleviate the discomfort.*

*However, if there are no other representations available (including that specific representation of the environment) to counteract the predictive error, its intensity and capacity to destabilize and unbalance the patient will be much greater. The ultimate prediction, such as “He is calling me a fool,” will acquire an absolute certainty. In this case, the patient’s attempt at top-down regulation poses a higher risk of impacting the environment in an unregulated manner. For example, he might verbally aggress the therapist to “take back” what was said or abruptly freeze and cease his collaboration in the session. Such responses would generate an intense and unexpected predictive error for the therapist, which will test his representational capacity, closing the circle in which both the patient and therapist act as emitters and receivers.*

These mutual regulation cases, where attempts to regulate one another become destabilizing factors, highlight the significance of the dyad in psychotherapy.

Tronick’s mother-infant model of dyadic states of consciousness, which can be expanded to the therapist-patient dyad, recognizes the importance of dyadic systems. These systems are constituted and regulated from the individual to the mutual level and vice versa (45, 46). Talia’s research on patient and therapist attachment patterns points in a similar direction, showing the bidirectional influences between both members of the dyad and the influence each has on the other, thus facilitating or altering the patient-therapist relationship (47–49). In this line, Schilbach and other authors (50, 51) propose a second-person neuroscience, arguing that to understand the mechanisms of social cognition and mental disorders, we must focus on the human dyad, since studying isolated individuals or experimental conditions lacks ecological validity, showing that when humans interact face-to-face, mental processes distinct from those of the individual or experimental condition are set in motion.

This perspective can also be applied to psychotherapy. Any psychotherapy is a process of joint attention and intervention on the patient’s mind, focusing on different aspects depending on the psychotherapy model’s framework (cognitive and affective contents, mental processes, etc.). However, it also involves an encounter with an equal “other,” requiring attention to what happens between the two individuals and the level of adjustment and coordination that occurs between them. Therefore, psychotherapeutic progress will require a prior, or parallel, movement of adaptation between both members—the constitution of a reliable dyad that operates within a tolerable margin of predictability, where both individuals must become co-dependent agents to maintain the dyadic relationship.

When an individual struggles with effective self-regulation, such as being dysregulated emotionally or experiencing disorganized thoughts or perceptions, their ability to collaborate in dyadic regulation is compromised (52, 53). This is particularly evident in patients with evolved SSD and serious mental disorders, who face greater difficulties in both internal and external regulation. In these cases, the therapist must take on a more active role in supplying and reinforcing stability within the dyadic system externally (52, 54).

However, there are even more challenging limitations when working with serious mental disorders. There are situations where the patient’s emotional intensity exceeds the therapist’s therapeutic capacity or when the patient’s relational patterns or psychic contents are incomprehensible to the therapist. Such situations will affect the therapist’s mind and saturate their predictive capacity, thereby exhausting their reflexivity and ability to maintain a therapeutic role. In predictive terms, the therapist repeatedly experiences excessive predictive errors, having difficulty minimizing them and leading to their own instability. This instability poses risks for both the therapist (in terms of acting, e.g. in a teleological way, getting overinvolved personally and materially in session) and the patient (in terms of potential iatrogenesis, e.g. inadequate assessment and adding chronically a drug in the patient’s treatment plan when it was just an acute event).

Studies on dyadic facial expressions (53, 54) support these two positions. They demonstrate that in unsuccessful psychotherapy treatments, therapists become overly involved in patients’ interpersonal patterns. However, they also highlight that in patient-therapist dyads, the therapy’s outcome is not directly related to the patient’s facial repertoire or expressive instability, but to the therapist’s ability to deal with them and avoid being involved in complementary patterns, which can be hypothetically linked to their capacity to “tolerate” predictive surprises.

When appropriately handled, these mismatches can serve as catalysts for change in therapy, stimulating the patient’s sense of agency (55). A similar assumption can be observed in discussions about infant self-development. Tronick (56) suggests that within the mother-child dyad, the ability to repair mismatches and dysregulations will be the highest expression of a robust sense of agency. This reinforces the individual’s capacity to continue expanding their capacity to control the environment and themselves. More recently, within the framework of the free-energy principle and mathematical paradigms, Tschacher (57) and Connolly (58) emphasize the importance of a “chaotic” mental process in therapy (as opposed to a deterministic one) where the instability and uncertainty generated by the therapist and his interventions, are critical in psychotherapy. This chaotic process, beyond the patient’s typical and rigid predictive patterns, is viewed as a necessary step for therapeutic change, where the patient experiences a broadening of thoughts, emotions, and behaviors to move them out of their deterministic priors.

However, a crucial question arises: how much mismatch (“chaos” or predictive error) can a patient tolerate? Connolly (58) addresses this point by stating that “there are clearly situations where instability is either undesirable or potentially harmful, in which therapeutic activities that activate chaotic processes should be avoided or at least mitigated or compensated for… such is the case of psychotic disorders.” Based on our experience, excessive predictive error in a patient, whether due to high demands or the patient’s limitations, can push him towards a representational leap for which he may not be prepared. This can increase the risk of disconnection from or collision with the environment, e.g. to ask for a cognitive challenge to a patient when his eyes are looking at the therapist but his mind appears absorbed in the inner voices he is hearing, or trying to validate empathically a patient presenting with psychopathological phenomena like thought insertion or thought stealing, which can produce a strong paranoid reaction.

In this regard, MBT has shown to be a safe approach when working with serious mental disorders (21, 59–61), monitoring the patient’s mental processes and fostering a trusting (predictable) dyad by adjusting its interventions to the patient’s mentalizing capacity at each point. But when treating evolved SSD this can become more difficult, given the absence or the alteration of external references to identify patient’s mental process (e.g. think about blunted affect, altered facial mimicry, not conversational turn-taking or not attentional correspondence). To resolve this questions our interventions in the therapeutic dyad will be guided by Gergely’s work on the development of the self as an agent.

## 3 Development of self agency and mentalizing

In the past two decades, developmental psychology and neuroscience research have shed light on the significant role of dyadic regulation in human development and psychopathology. Fonagy (62) describes how the mother’s regulation of the infant’s mental space within the context of attachment serves as a temporary external regulatory agent during development, assisting in the formation of the mental representations of the child through a process of resonance with the mother’s own mental apparatus.

Within these dyadic contexts, infants achieve crucial milestones that strengthen their sense of agency, such as self/no-self differentiation (37, 63, 64) and the progressive development of more complex mental representations of themselves and others (62). This process is mediated through dyadic infant-self tailored mirroring responses. These include time and emotion contingency (for infant referencing), marked responses (to differentiate the partner’s authorship from the infant’s own), and ostensive cues (to attract the infant’s attention with specific referencing for him). These interactions facilitate partial predictability and co-dependency processes (65), strengthening both the sense of agency and ability to make accurate attributions about the intentions of others and, with time, constancy and rupture-and-reparation processes (mismatch and match). Together these processes will lead to the development of a more robust bidirectional self-regulating dyad for both members, fostering a second-person perspective (63).

To facilitate this progression, Gergely proposes different stages in the development of the self and agency (62, 66), with the final stage being a mentalizing agent with representational and autobiographical capacities, capable of understanding one’s own behavior and that of others in terms of intentional states of mind.

In our hypothesis to identify these stages and their characteristics will be crucial for our therapeutic purposes. They reveal which agent capacities are at work within the dyad at each point, indicating the contributions each member needs to make to achieve a stable regulated and trusting dyad. This understanding guides the therapist in recognizing the patient’s agency limitations and determining the extent of their intervention required to repair, support, or challenge the patient’s agent capacities.

The first stage Gergely proposes is the self as a physical agent (66), relying on proprioceptive and perceptual sensory perfect contingencies to detect one’s own body in space and its orientation. It determines the body-physical environment boundaries. At this level, there is no place for an equal other; instead, the other is perceived as an environmental object^1^.

The next stage is the social agent, where the individual engages with another person, recognizing and interacting with them in an affective and intentional manner, engaging in co-dependent acts such as seeking the other’s attention or engaging in turn-taking behaviors. The transition from physical to social adaptations is mediated by a shift in preference from perfect contingencies, typical of the physical world, to less-than-perfect contingencies (62, 66), typical of the social world^2^.

In the subsequent stage (66), the teleological agent, the individual considers themselves and others as intentional agents, interpreting their behavior as driven by rationality and creating efficient-based causal explanations based on the goal of the action. In this stage, there is place for joint attention and the possibility to think and talk about intentions after action execution, although not yet in mental terms, and without considering the other’s intentions as distinct from one’s own.

This leads to the intentional mental agent stage (66), where the self and others are recognized as agents with intentional “invisible” mental states (needs, desires, feelings, beliefs) that precede action execution (or not-execution). Recognizing these mental states enables individuals to induce, share, or modify them in each other.

Finally, in the representational agent and autobiographical self stage (66), individuals are capable of representing and recognizing a stable autobiographical sense of self and others beyond their eventual mental states. This stage involves the understanding of false beliefs and executive control, providing abilities for more adaptive social functioning.

Usually in the MBT foundational technique for adults we will work on teleological agent stage or superior, leading from pre-mentalizing modes to a deeper mentalizing understanding of the self and other, as well as a more constant and integrated view of self and others (59).

However, in our hypothesis, for patients with evolved schizophrenia and SSD, we will need to extend attention and intervention to the first two stages, physical and social, the protomentalizing stages, in which the limited patient’s self-agency capacities are strongly compromised to reciprocally deal with another self. This will affect the therapist, potentially causing difficulties in understanding both members of the dyad.

## 4 Mentalization-based approach for SSDs

MBT is a therapy that focuses on the process rather than achieving representational coherence and integration. Its aim is to restore the ability to mentalize, when is lost due to stress, attachment, and non-mentalized affects, and it involves a continuous movement between stabilizing and stimulating mental processes (59, 67).

The mentalization based approach for evolved Schizophrenia spectrum disorders introduced here follows a similar approach to MBT (59), but rather than aiming to restore or consolidate the patient’s mentalization (as in the case of MBT for personality disorders), the goal is to create, repair, supply, or support basic mental functions and phenomena (such as attentional, perceptual, emotional and reflective abilities) so that they can later be aggregated or re-aggregated to configure progressively more complex representations.

This is an approach that complements MBT for Psychosis (MBT-P) (9, 21–29, 31, 61), allowing the inclusion of those more severely affected patients who have difficulties in psychotherapy with reciprocal dialectical work, due to impairments in their attentional, perceptual, cognitive or affective capacities.

Technically, it has two main adaptations: changing the affect focus for a dyadic-agency focus, and to be guided by the development of the patient’s agency in order to work with pre-mentalizing modes of functioning.

To achieve this, it expands its scope of work to protomentalizing self agency functions, those regarding physical and social agency stages in Gergely’s theory (62, 65, 66). By doing so, the aim is to create a predictable environment for the patient during sessions, initially allowing them to feel socially secure with the therapist, so that subsequently, they also perceive the therapist and the conveyed contents he transmits as reliable.

A primary therapeutic factor in therapy (and in human development) is the development of epistemic trust, wherein individuals accept certain contents or information solely because the transmitter has proven to be trustworthy (68, 69). These authors propose that for another individual to generate this trust, they must first demonstrate contingency toward the individual’s mental state and respect for their agency capacity (69).

In our case, we hypothesize that a prerequisite to developing this epistemic trust in therapy —to accept the information and attentional proposals offered by the therapist to the patient, is to establish this kind of environmental trust, a predictable social environment that allows the patient to rely in the therapeutic dyad.

Dyad and self-agency are the two fundamental structures of this approach. The dyad serves as the mental workspace for both the patient and the therapist, while agency, as expressed within the dyad, serves as a guide to indicate which functions are being utilized, the degree of adjustment, and the establishment of the patient’s self-boundaries in a Markovian sense. Therefore, the working area of our proposal will be focused on self-boundaries.

Typically, aside from reflexes, affect is considered the primary driver of agency expression in human beings (70). We recognize agency through emotional expressions triggered by stimuli (such as rejection, anger, sadness). However, in the case of schizophrenia, these expressions of agency may be abnormal or challenging to identify (e.g., blunted affect, inhibited behavior, fixed or absent facial mimicry). In such cases, the clinician must observe other expressions of agency in the behavioral, paraverbal, or verbal dimensions within the therapeutic dyad. These expressions can range from subtle indicators (e.g., psychomotor restlessness, lack of turn-taking consideration, implicit rejection of topics raised by the therapist) to particular relational offers (e.g., suspicion, distrust, inadequate familiarity) or to more disorganized or unusual behaviors (e.g., symbiotic or submissive merging behaviors, echolalia, oppositionism). Importantly, our hypothesis emphasizes that the sense of agency is not determined by the patient’s intention behind his actions (something that is impossible for us to know). Rather, agency is attributed based on the impact the patient has on the therapist, who acts as a proxy for the environment. This is the true significance of dyadic agency.

### 4.1 Working on self agency actively or passively

Using dyadic agency as an indirect (in our case, tentative) guide to the individual’s level of adaptation to their environment will accompany the therapist throughout the therapeutic process and determine which stage of self-agency development is being expressed and what type of intervention can be used. The therapist will then intervene based on a main pair of premises we enunciated before: to try externally to minimize predictive error for the patient or inducing tolerable predictive error. Or, in other words, stabilizing the dyad (consolidating the patient’s agency -passively for the patient) or introducing mismatches (actively stimulating patient’s agency).

As hypothesized, considering the amount of “chaos” or predictive error the therapist sends to the patient is crucial in therapy. The clinician will be guided by the self-agency developmental stage (physical, social, teleological, mental, or autobiographical) to identify where the patient’s dyadic agency is failing. Therapist will then proceed to stabilize the dyad at that level and, after this, to start trying to progress to a more demanding agency.

Stabilizing dyad. Stabilizing a dyad towards a lower stage can be very challenging for the therapist. In MBT this is often achieved through employing empathic validation, guided by the affective resonance that the patient provokes in the therapist. But in advanced SSD, the therapist will frequently encounter predictive errors that can be intense (e.g., not a clear affect to resonate with, patients with incomprehensible speech, bizarre relational offers, rejection of linking). This entails a significant risk of the therapist losing mentalization, leading to modes such as pretend mode (e.g. therapist can hypermentalize -an uncontrollable overproduction and attribution of intentions), psychic equivalence (being certain about what is happening in the patient’s mind), or teleological functioning (the therapist acting instead of mentalizing), which perpetuate dysfunctionality in the dyad. To stabilize the dyad, supporting agency from the outside, the therapist must move in reverse order towards self-agency development, reaching a level where the dyad feels safe. This may involve shifting from autobiographical issues to mental ones or from mental to concrete ones, or from concrete to attentional or relational ones. In our case, when working with evolved SSD the most demanding work occurs when operating at lower levels where joint attention is impaired, and the therapist has fewer references to work with. At these levels, the therapist’s task is to simplify interactions through marking and ostensive interventions or to “clean” the sensory “noise” therapist emits by employing different strategies such as lowering sensory stimuli (voice, prosody, body attitude, timing pressures), being clear in speech, avoiding contradictory facial emotional expressions, and narrating their own behavior as it unfolds.

Stimulating dyad. On the other side, to gradually provoke increasing agency demands, the therapist will move upwards tentatively step by step, in line with the stages of developmental self-agency. Moving towards more advanced levels will be easier for the therapist as the patient will demonstrate progressive agency capacities, aiding in dyadic regulation, and the contents both share will be more accessible for language and accurate transmission.

### 4.2 Working tolerating prementalizing modes

When working on MBT with personality disorders, another key task is to deal with prementalizing modes as soon as they are detected, to recover patient’s mentalizing. However, in cases of these evolved and chronic conditions, as mentioned earlier, where there are symptoms and altered neurocognitive, social cognitive, or metacognitive functions, our aim will be to consolidate and strengthen the patient’s demonstrated higher agency level. In such cases, the therapist will prioritize a dyadic more predictable interplay over progress attainment. This means that the therapist may need to tolerate and even purposefully work in non-mentalizing modes, refraining from demanding what the patient cannot achieve. For example, functioning in pretend mode and using metaphors or personal and fantasized events involving third parties or oneself may be useful if they facilitate the appearance of mental contents (affect, intentions…) in the narrative, regardless of their mental accuracy.

## 5 Clinical case

X is 36 years old. He has been diagnosed with schizophrenia and lives with his mother, avoiding social interactions. In therapy sessions, he exhibits logorrhea, excessive and rapid speech, which in his case is not indicative of a manic syndrome, as his thought maintains a normal speed. He leads an orderly, even rigid, life. He can wait placidity in the waiting room before sessions and is disciplined with the instructions given to him. In sessions, he talks about several things that his mother asks him (such as to run errands). However, his logorrhea prevents him from letting his partner talk. Whenever the therapist is about to speak, X abruptly resumes speaking about unrelated topics, blocking the therapist’s turn.

In terms of relational offer, the patient recognizes the therapist and initiates an attentional offer by discussing errands and tasks assigned by his mother. X outwardly appears calm and cooperative, providing an opportunity for the therapist to engage him on a mental agency level (e.g., “how do you feel when your mother asks you for these errands”) or a teleological level (e.g. “how do you organize yourself to manage this”. “how do you organize this with your mother”). However, due to the lack of true joint attention, genuine conversation becomes unattainable. There is an attentional offer (an initiation of joint attention), but not possibility for a *response* of joint attention.

This scenario can easily lead the session to resemble a monologue, where the therapist, after a while, eventually concludes with recommendations related to behavior or medication. This outcome would result in two separate monologues or, even worse, the therapist imposing their own beliefs about what is important for the patient. Both outcomes represent alienated forms of communication that deviate from fostering a genuine therapeutic relationship.

However, from the perspective of dyadic agency, the therapist acknowledges that he is unable to speak. Therefore, therapist reduces his own required dyadic agency to a lower stage, focusing on the simple recognition of the presence of an “other” attempting to interact with him (Gergely’s self as social agent). Then therapist gently moves his hand in a stopping motion and says, “Hey, I cannot talk.” Through these soft ostensive cues, the therapist aims to attract the patient’s attention while delivering a brief and unambiguous message—a self-revealed state—to reduce sensory complexity at a physical agency level.

Although X initially paused, he continued with his logorrhea, prompting the therapist to maintain this intervention style throughout the session. The therapist repeatedly sent messages such as, “Hey, I want to talk, but I can’t,” or “I’m listening to you, and I don’t want to interrupt, but I would like to say something when you finish.” Finally, at the end of the session, the therapist said, “Today, I didn’t talk. Maybe next session”.

In the subsequent session, X resumed his logorrhea, and the therapist continued with this intervention style. However, at one point, X fell silent and expressed, “I feel bad when I have to listen to you. Serious insults come to my mind when I’m silently listening, and it makes me feel bad. My loud voice distracts me from them”.

This abrupt revelation elevated the dyadic agency to a higher level, with X offering attention to his own mental state and revealing that his behavior is caused by an emotional drive.

Upon hearing this, the therapist appreciated the revelation and marked its impact on him (“Oh, now I understand you, thank you for telling me”). The therapist then proceeded to test X’s response to joint attention at a mental level, saying, “We will need to address this issue the next time you speak over me,” “I’ll be attentive, but I’ll need your help to determine if I’m being helpful or if I’m annoying you.” X agreed to this approach. In the following sessions, the therapist and X began exploring ways to address this issue during sessions and outside, as well as discussing the impact it had on his interpersonal relationships.

## 6 Discussion

The mentalization-based approach for evolved SSD outlined here aims to be useful for working with patients when it’s not possible for the therapist to apply MBT foundational technique, that is, when it’s not possible to generate joint attention, joint intentionality, empathic connection, when there are relevant alterations in the patient’s agency capacity, or when the patient remains in non-mentalizing modes chronically. For this purpose, our main proposal is to shift the affective focus to the dyadic agency focus.

The theoretical support for these adaptations is found in Friston`s free-energy minimization and predictive coding and the second-person paradigm, which allow us to understand the type of relationship generated in an unpredictable dyad. Bayesian and free-energy explanatory models have been proposed for psychotherapy and for understanding schizophrenia psychopathology. Some interesting developments implementing these models in psychotherapy have been made (41, 57, 58). In the context of SSD, these models have been utilized too to explain the illness (71) or to understand hallucinations and delusions as disturbances in the patient’s predictive system (40, 72–74). Furthermore, extensive research has examined the role of agency capacities in patients with this diagnosis from various neurocognitive and neurophilosophical perspectives (75–78). Studies in second-person neuropsychiatry emphasize the importance of the dyad in understanding pathology and psychopathology, shedding light on etiology, symptom maintenance, and treatment possibilities (79, 80). Additionally, research about dyadic facial expressions have proven the impact of schizophrenia on the emotional expression of the dyadic partner, who must contend with the patient’s dysregulated emotional register, leading to a downward adaptation in their own facial emotional expression (53, 81, 82).

The other key point of our proposal is to rely on Gergely’s work on the development of the self as an agent, which will allow us to work on protomentalizing levels of self-agency while providing us with a staging that will serve as a guide to safely advance in the dyad, supporting or stimulating it to the extent that the dyad can tolerate.

In doing so, our aim is to strengthen the patient’s agency and enhance their adaptation to the environment through the establishment of a secure bond with the therapist, which allows both parties to work on the patient’s more adaptive self and self-and-other comprehensions.

The goal is for these improved understandings and the therapeutic relationship itself to be transferable to other professionals and the broader social network, aligning with the typical objectives of MBT (69, 83).

Although symptom reduction and improvement in overall functionality are not primary objectives, an improvement in them is envisaged in parallel with the creation and consolidation of the reliable dyad. Another important issue is that the establishment of a trustworthy dyad, especially with isolated patients, will also give us the opportunity to supervise global well-being and address tertiary prevention issues, considering the excess morbidity and mortality of these patients (84, 85).

The main and most obvious limitation of our proposal is the lack of empirical data supporting its utility. Although grounded on research, our approach is heuristic and needs to be evaluated. Currently, we are in the process of manualizing it and hope to conduct an initial pilot study in the short term.

Another relevant issue pertains to the position of the therapeutic approach discussed here in relation to other treatments. We chose the term “approach” because our proposal is not a treatment in a full sense, but rather a complementary approach to treatment for those patients with severe mental disorders to establish a reliable therapeutic connection which may place them, concurrently or subsequently, in a position to undergo further psychotherapeutic treatment. Similarly, it will be usually complemented by social interventions and pharmacological treatment and may also benefit from other MBT approaches such as group interventions, MBT with families (86) or AMBIT (Adaptive mentalization-based integrative treatment) (87). The supervision of the therapist will be necessary, as usual in MBT, due to the demanding mentalizing job when working from a dyadic agency approach.

Another aspect for which we consider it an approach rather than a treatment is the lack of defined length of intervention. As we have seen, our proposal can serve as a preliminary stage before MBT-P, but it also functions as a way to engage with patients in need of chronic adaptations. In general terms, those patients with more favorable conditions, such as those cases marked by environmental conditions, with acute unfavorable life experiences disabling their sense of agency, may require shorter intervention periods. These individuals may find it easier to establish link with mental health professionals and to experience mentalizing growth. On the other hand, patients with a greater biological burden, more severe neuropsychological deficits, or disorganized attachment styles and relational difficulties will necessitate longer therapies, and more intensive work on establishing connections and adapting to their environment.

We believe that the presented mentalizing approach holds promise for future research and opportunities in the treatment of patients with advanced schizophrenia and serious mental disorders. Furthermore, it provides mental health professionals with perspectives and tools that can assist them in developing deeply respectful and long-term relationships with individuals with seriously altered functionality who face significant communication challenges and struggle to adapt to their environment.

## Data availability statement

The original contributions presented in the study are included in the article/supplementary material. Further inquiries can be directed to the corresponding author.

## Ethics statement

Written informed consent was not obtained from the individual(s) for the publication of any potentially identifiable images or data included in this article because therapeutic relationship and paranoid symptoms could be worsened. All the original personal data that appear in the case have been changed or modified (sex, age, lifestyle, family members) or omitted (temporal and geographical location), making it impossible to identify this person.

## Author contributions

PS contributed to conception of this article. All authors contributed to the article and approved the submitted version.

## Footnotes

1 We can find alterations at this level for example in the way they could ignore or not to consider their own body care or needs, like pain, temperature regulation or hunger, or first rank psychopathology like own body boundaries alterations, e.g. not to control a part of the body (or being controlled externally), hearing outside what it is inside, “hear” a thought, or perceive their thought is visible outside, to name a few.

2 Alterations at this level can include, problems with joint attention (not initiating, not responding, refusing), altered relational offer (in affect expression or affect responsiveness, querulant, negativistic) or dialectical communication issues (altered turn-taking, mutism), to name some.
